# A qualitative assessment of experiences and perception during pre-admission for rotator cuff repair surgery

**DOI:** 10.1186/s12891-023-06350-9

**Published:** 2023-04-03

**Authors:** Umile Giuseppe Longo, Nicolò Panattoni, Sergio De Salvatore, Anna Marchetti, Maria Grazia De Marinis, Vincenzo Denaro

**Affiliations:** 1 Fondazione Policlinico Universitario Campus Bio-Medico, Via Alvaro del Portillo, 200, Roma, 00128 Italy; 2 Research Unit of Orthopaedic and Trauma Surgery, Department of Medicine and Surgery, Università Campus Bio- Medico di Roma, Via Alvaro del Portillo, 21, Roma, 00128 Italy; 3 Department of Biomedicine and Prevention, University of Rome Tor Vergata, Via Montpellier 1, Rome, 00133 Italy; 4 Research Unit Nursing Science, Campus Bio-Medico University, Via Alvaro del Portillo 21, Rome, 00128 Italy

**Keywords:** Rotator cuff tear, Rotator cuff repair, Experiences, Arthroscopy, Qualitative research

## Abstract

**Background:**

Rotator Cuff Related Shoulder Pain (RCRSP) is the most common type of shoulder pain and the most disabling common symptom in people with Rotator Cuff Tear (RCT). The patient’s point of view concerning health status has become important in decision-making procedures and has therefore been considered a possible criterion standard for assessing treatment efficacy. The study aims to explore patients’ experiences and perceptions during pre-admission for Rotator Cuff Repair surgery.

**Methods:**

A qualitative descriptive study was conducted using a phenomenological approach based on Husserl’s philosophical perspective. A consecutive sample of twenty RCT patients awaiting repair surgery agreed to participate and was interviewed until the information was saturated. None of the patients enrolled was lost during the data collection phases. Data were collected through open-ended interviews between December 2021 and January 2022. The credibility, reliability, confirmability, and transferability criteria of Lincoln and Guba have been adopted to guarantee the trustworthiness of the results. The data analysis was conducted according to inductive content analysis.

**Results:**

Four main themes and sub-themes related to each have been identified from the phenomenological analysis. The major themes were: (1) Pain changes lifestyle habits, (2) Pain control requires specific strategies, (3) Suffering turns time into waiting, (4) Waiting for Surgery between trust and fear.

**Conclusion:**

Investigating patients’ experiences and the emotional impact of Rotator Cuff Tear facilitates the development of specific educational and therapeutic strategies to improve care and post-intervention outcomes.

## Background

Rotator Cuff Tear (RCT) is one of the most common shoulder disorders in people between 60 and 80 years [1, 2]. Rotator Cuff Related Shoulder Pain (RCRSP) is the most common type of shoulder pain [3] and the most disabling symptom common to people with RCT [4]. Patient’s with RCT experience persistent pain, different for each individual, and can lead to disability and reduced health quality [5]. The most significant complaint of patients is increasing night-time pain and disrupted sleep [6]. Therefore, improving sleep quality in patients with rotator cuff tear may be a significant factor for healing surgical intervention [1, 2, 6].

In orthopaedic patients, pain is usually assessed as current pain severity on a Likert-style scale or Visual Analog Scale (VAS). However, these scales have limitations since they do not consider the complexity of the individual’s pain experience [7]. Therefore, it is necessary to understand further patients’ experiences, perceptions, values, emotions, beliefs and coping strategies and the influence of all these determinants in the self-management of the pathology [8]. In addition, the patient’s point of view concerning health status has become important in decision-making procedures. It has been considered a possible criterion standard in assessing treatment efficacy [9].

Scientific evidence also focuses on evaluating preoperative psycho-social factors as they could influence functionality, disability and perceived pain in patients with RCT [2]. Besides pathoanatomical structures, neurophysiological rules also provide for the patient’s pain experiences [10, 11]. In a study by Lau et al., patients with rotator cuff tears showed preoperative depression and/or anxiety [12]. Hessburg et al. indicate that patients with preoperative depression demonstrate improvements in physical function, pain interference, and even depressive symptoms following arthroscopic Rotator Cuff Repair (RCR) [4].

Currently, the literature investigating patients’ experiences, perceptions and points of view with RCT is poor, and many aspects of the disease are analysed only by quantitative scores. However, the inter variability of the pain perception could alter the results.

Qualitative research could represent the best choice for understanding patients’ needs and could improve the use of targeted and personalised therapeutic strategies [9, 13, 14]. In contrast to quantitative research, qualitative research focuses on “why” and “how” questions and seeks to generate thoughts about a phenomenon through an in-depth understanding of the perspectives of a small number of individuals experiencing the phenomenon [15, 16]. In addition to interventions and therapeutic strategies, qualitative research finds strength in returning essential information to elaborate patient educational strategies. Moreover, patient education and coping skills reflect better outcomes regarding self-efficacy and self-care, reduction of unnecessary treatment, and significant cost-benefit improvement [3].

Therefore, it is necessary to conduct studies that assess the qualitative perception of pain and functionality in RCT, obtaining a different point of view on the topic as it could allow for more personalised therapies.

The study aims to explore patients’ experiences in pre-admission for Rotator Cuff Repair surgery.

## Materials and methods

### Design

A qualitative descriptive study [17] was conducted using a phenomenological approach based on Husserl’s philosophical perspective [18].

This phenomenological approach was chosen as it allows for the acquisition of content-rich experiences, perceptions, emotions, judgments, perspectives and visions of the world of the patients involved in the study [18]. Phenomenology returns to the phenomenon explored as it is perceived and interpreted by the person involved [19]. The descriptions are detailed, expressed in a language that can easily be understood by people who experience the phenomenon and offer the opportunity to acquire internal knowledge, observe the world with the sentiment of those who describe it and use this knowledge to influence the interventions [20]. Giorgi’s descriptive phenomenological method was used to process and analyse the text generated by the participants’ narratives. This method involves a minimum of five basic steps: (1) collection of verbal data, (2) reading of the data, (3) breaking of the data into parts, (4) organisation and expression of the data from a disciplinary perspective, and (5) synthesis or summary of the data for purposes of expressing the structure of the phenomenon [19].

### Participants

A consecutive sample of twenty RCT patients awaiting repair surgery agreed to participate and was interviewed until the information was retrieved. None of the patients in the sample was lost during the data collection phases, and all twenty enrolled patients completed the interview. After each day of interviews, a saturation grid was used to define data saturation, hence the term of enrolment, indicated by the absence of meaningful information provided by new respondents [21].

To ensure the adequacy and quality of the sampling strategy [22] patients were recruited according to the following inclusion criteria: (1) adult patients (age < 18 years), (2) patients with complete rotator cuff tear during pre-admission for repair surgery, (3) consent to participate in the study. Two orthopaedic surgeons specialising in shoulder arthroscopy assessed RCTs by preoperative magnetic resonance imaging (MRI) and clinical examination. Only patients with Goutallier grade 2 [23] and Patte stage 2 lesions were included in our study [24]. Patients not undergoing surgery or other types of shoulder pathologies were excluded. All the participants received all the necessary clarifications on the research objective and the interview modalities.

### Setting and data collection

Data were collected through open-ended interviews between December 2021 and January 2022 at the Department of Orthopedic and Traumatological Surgery of the Campus Bio-Medico University Hospital of Rome, Italy. The interviews were scheduled according to the pre-admission list for surgery. All interviews were conducted in hospitals (e.g., a meeting room or clinic). The interviews lasted 5 to 15 min and were digitally audio-recorded and transcribed verbatim.

Before starting the interview, the main themes of the study were explored, the researcher encouraged the interviewees to narrate freely in response to the questions, and the consent was signed.

The questions were designed to allow respondents the flexibility to direct the flow of the conversation and structure it to ensure critical topics were addressed.

The following questions were asked: Can you tell me about your health problem? What changes (physical, psychological, emotional, etc.) did it bring about in your life? What are your fears (emotions/thoughts) about the future of your illness? How do you sleep? How is the quality of sleep? Would you like to add more?

All twenty participants answered the abovementioned questions in a free-form response mode.

### Data analysis

The data analysis followed the steps outlined by Giorgi’s descriptive phenomenological method [19] and was conducted according to the analysis method of the inductive content analysis method [25].

For the first step, the researcher who conducted the interviews performed the collection of verbal data, while a second researcher transcribed the verbatim, noting pauses, gestures and emotions (pointed out in the field during the interview) to reflect the meaning of words and preserve the accuracy of verbal data collection. During the second step: reading the textual data in its entirety, the researchers kept an open mind to get a general sense of the information gathered rather than trying to thematise any aspect of the descriptions. In the third step: splitting the data into parts, once each researcher gathered a general sense of the material, the data was divided into two parts, and the text was carefully reread line by line to identify the “units of meaning”, defined as distinct units that express an autonomous meaning [19]. In the fourth step: organisation and expression of data from a disciplinary perspective, each of these units of meaning was examined and reorganised to make its disciplinary value more explicit and expressed in terms relevant to the clinical context. In the fifth and last step: the synthesis of the data to define the structure of the phenomenon, the units of meaning were grouped by compressing similar ones into more extensive subcategories in an attempt to synthesise the meaning of the data and describe the structures concerning various manifestations of an essential identity. In the abstraction phase, the subcategories with conceptual and semantic similarities were grouped into generic categories. The structures were intended as essences, and their relationships were made explicit through themes and sub-themes.

A senior researcher experienced in qualitative research oversaw all phases of the analysis, and the research team discussed all discrepancies until a unanimous agreement was reached.

For this study, the researchers did not use specific qualitative data analysis software, preferring the transcripts on grids and tables built for group analysis and discussion of each step.

### Trustworthiness

To guarantee the trustworthiness of the results, credibility, reliability, confirmability and transferability, the Lincoln and Guba criteria were adopted [26]. To ensure credibility, we continued sampling with data collection until it was saturated. Reliability was guaranteed through the triangulation technique, which involves the participation of two or more researchers in the same analysis to provide both confirmations of the results and different perspectives, adding breadth to the study phenomenon and multiple conclusions [26]. Confirmability was ensured through audit trails, in which two researchers not involved in data collection and analysis reviewed the survey process and resulted in providing that the data supported the results. Finally, the transferability was demonstrated through the description of the socio-demographic characteristics of the participants and an accurate and detailed description of the methodology, the research process and the results.

### Ethical considerations

The study was conducted according to the Declaration of Helsinki [27]. It was approved by the Institutional Review Board of Campus Bio-Medico University of Rome (COSMO study, Protocol number: 78/18 OSS ComEt CBM, 16/10/18).

Eligible patients were contacted by telephone during their pre-admission day. They received verbal information on the study’s purpose, the interview modality and the need for a digital audio recording. They were also informed that their contribution to the study would be voluntary and free and that the data collected would be analysed and reported in a way that ensures confidentiality. All participants then signed informed consent for this study’s adherence before data collection. Anonymity was guaranteed through an identification code during the transcription of the interviews and the entire data analysis process, ensuring that no single participant could be traced in the reporting study. Participants were also told they could withdraw from the study at any time.

## Results

### Participant characteristics

A sample of twenty patients, 12 male and 8 female, with a mean age of 58.4 years, in the pre-admission phase for RCR surgery, agreed to participate and were interviewed.

The participants’ clinical and demographic characteristics are described in Table 1. It is a sample younger than the mean of patients which experimental RCT disease, as reported in the literature. Aetiology is a prevalent traumatic type in this population, often during the work schedule. This is demonstrated by the differentiation between working activity (16 patients) and not working (4 patients).

**Table 1:** Demographic characteristics of participants (N = 20)

Patient characteristics	n
Age, mean (range)	58.4 (46–70)
Gender	
*Male*	12 (60%)
*Female*	8 (40%)
Affected side	
*Right*	16 (80%)
*Left*	2 (10%)
*Bilateral*	2 (10%)
Dominant arm	
*Right-handed*	19 (95%)
*Left-handed*	1 (5%)
Symptoms’ duration (months), median (range)	9.5 (1-120)
Trauma	
*Yes*	10 (50%)
*No*	10 (50%)
Working	
*Yes*	16 (80%)
*No*	4 (20%)

### Phenomenological analysis findings

Four main themes and sub-themes related to each have been identified from the phenomenological analysis [25, 26]. Table 2 summarises the results. The themes below are supported by the words extracted from the interviews, identified by patient code (P1-P15), sex (M = male, F = female) and age, e.g. (P15, M, 59 yrs.).

**Table 2:** Themes and sub-themes

	Themes	Sub-themes
Themes and sub-themes	Pain changes lifestyle habits	- Restriction or inability to move
		- Impaired sleep quality
		- Dependence on basic needs
	Suffering turns time into waiting	- The underestimation of the problem
		- Negative feelings generated by pain
		- The wait for a solution
	Pain control requires specific strategies	- Adoption of autonomous interventions
		- Adoption of pharmacological/medical prescriptions
	Waiting for surgery between trust and fear	- Optimism and confidence for arthroscopic surgery
		- The fear of not solving

#### Pain changes lifestyle habits

Pain is undoubtedly the dominant symptom with pain in the shoulder and arm, common to all patients from the onset of the disease and is responsible for the most significant changes that patients experience. The beginning of the disease can be acute and traumatic following falls on the ground, loss of balance, direct trauma on the affected arm, or secondary to other pathologies such as osteoporosis or repetitive work activities. According to this distinction, the pain can be perceived as very acute and excruciating, like a stab or a sensation of continuous discomfort.

In any case, it is a persistent pain at a precise point that necessarily affects normal daily activities, has a negative impact even in activities during work, and upsets the hours of sleep.

“(…) the world changed overnight.

(…) I cannot do daily activities, and consequently, problems arise” (P15, M, 59 yrs.).

Therefore, this theme has brought out three sub-themes described below: restriction or inability to move, poor sleep quality, and dependence on others for basic needs.

##### Restriction or inability to move

Most interviewees reported that pain generates severe limitations in arm movements (such as lifting or holding things) which they consider a very penalising event. To overcome the limitation, some claim to physically exert themselves and perform calibrated and studied movements to limit the pain.

“I can’t do many things anymore” (P1, F, 67 yrs.).

##### Impaired sleep quality

Interviews show the strong negative impact of RCT and related pain on sleep. Patients cannot sleep well at night; they sleep very little, suffering through the night. Sleep problems are different; some complaint of repeatedly waking up every time they move in bed. Others complain of not being able to fall asleep because of the pain. Conversely, some do not find an “analgesic” or comfortable position that relieves shoulder pain. In any case, the quality of sleep is impaired, it is judged to be just sufficient, and sleep is not regenerating, negatively impacting waking hours.

“It is awful [the quality of sleep], I usually sleep on one side and change sides often at night, and therefore the right side is penalised, and it is difficult to find the position to fall asleep at night, then naturally I collapse from fatigue, but often I wake up, or I wake up at night with shoulder pain” (P10, M, 51 yrs.).

##### Dependence on others for basic needs

The condition of pain and severe functional limitation implies the dependence on other people for assistance even in simple, everyday life. It highlights the need for help that these patients express. If carried out independently, daily activities require physical effort and sacrifice and are not carried out as they should be. The activities that need someone’s help are the simplest: getting dressed, washing, eating, simply fastening the bra, driving, cleaning the house, washing clothes, and putting your hair behind your ear.

Due to the condition described, patients perceive a state of incompleteness, insufficiency, incapacity, and suffering, and their desire is exclusively to regain all functionality.

“Lots of difficulties because when the pain becomes acute, I can’t even get my panties up, button up my bra, normal life things, take a plate from the dish drainer. So be addicted to so many things. It was awful” (P18, F, 54 yrs.).

#### Pain control requires specific strategies

All interviewees stated the need to adopt appropriate behaviour and pay attention to controlling pain. This allows them to continue to carry out their daily or work activities, albeit with limitations.

Patients’ strategies are the most varied and sometimes very personal, including adopting certain antalgic positions. They also resort to drugs or physical therapies depending on the doctor’s prescription.

“I find some relief even if the pain is always in the background” (P2, M, 46 yrs.).

##### Adoption of autonomous interventions

Each patient interviewed mentioned having adopted personal and autonomous interventions to cope with pain in the various phases of the day and to try to lead an everyday life.

The most adopted strategy is undoubtedly to help the sore arm with the other healthy one, particularly those affected by the dominant arm, help with the contralateral, or replace the use of the affected arm with the healthy one, learning new skills.

Some adopt positions that relieve pain during the day, such as extending their arms from the sofa and sleeping on the side of the painful shoulder at night.

Other strategies are inherent in the specific case. For example, one patient said she found relief in sandblasting; another interviewee bought special pyjamas to keep the affected shoulder warm, and another changed pillows until she found relief during the night.

“At the beginning, when I had the first symptoms. I even had to accompany the right arm with my left arm” (P11, M, 59 yrs.).

##### Adoption of pharmacological/medical prescriptions

Before deciding in favour of surgery, some patients also adopted different strategies following the prescriptions of the doctor they contacted. These prescriptions covered infiltrative therapy, pain-relieving drugs, and anti-inflammatory physiotherapy.

“I tried palliatives, anti-inflammatories, and physiotherapy” (P3, F, 55 yrs.).

#### Suffering turns time into waiting

This theme contains the thoughts, emotions, and feelings that accompany patients from the moment they are affected and discover the first difficulties related to pain but underestimate the problem believing that they can continue to move on with life until the waiting is filled with hope and impatience of the surgery, the only solution to the problem.

Plight and suffering are the factors that increase daily and project all hopes in RCR.

“I find the situation dramatic” (P13, F, 65 yrs.).

##### The underestimation of the problem

Generally, patients in an initial phase tend to need to pay more attention to the extent of the problem. They try to continue to carry out all their usual activities while neglecting the initial pain. This happens especially when the injury does not manifest in an acute and traumatic way, and the discomfort can be endured for months.

This initial underestimation also leads to postponing the intervention several times, believing it can be resolved without it.

“It started with small pains (…) at first they were very mild and then slowly they began to increase. Then they went in periods, that is, a month I had it, and a month they disappeared, and in winter, I noticed that this was when I felt it the most. And now the pain has become more intense “ (P14, F, 52 yrs.).

##### Negative feelings generated by pain

As mentioned, pain and functional limitations are common to most patients and are the manifestations that cause the most discomfort. In particular, the patients interviewed described pain from an emotional and psychological point of view, generating negative feelings and is described as suffering, a disaster, a mortification that creates nervousness and fatigue.

“Psychologically, it knocked me down” (P11, M, 59 yrs.).

##### The wait for a solution

Most interviewees say of arriving when it becomes inevitable to find a solution, to decide to face surgery: to make a choice. The decision to act is often postponed for fear of post-operative recovery, for personal accident causes, or hoping that everything will be resolved without any external intervention. In the moment of choice, their perception of time changes. You realise that time passes, and you must take action to improve your health. Underestimation turns into expectation.

“I can’t wait to be operated on” (P14, F, 52 yrs.).

#### Waiting for surgery between trust and fear

RCR is regarded by all respondents as a lifeline and generates mixed feelings. Common to most interviewees is the total trust placed in surgeons, their teams and the hospital. All the hopes of the patients are poured into the intervention: the success of the surgery, which will prevent the recurrence of this episode in the future and take them back to their previous life and the disappearance of pain.

“I hope everything is fine and the arm is fine again, as it was before.“ (P14, F, 52 yrs.).

##### Optimism and confidence for arthroscopic surgery

All the patients interviewed showed that they understood the need for surgery. The session with the orthopaedic surgeon during the pre-admission phase is crucial, as patients are informed about the type of arthroscopic surgery. This news brings positive feelings and much confidence. Despite their living conditions, patients perceive arthroscopy as low-impact and minimally invasive. This perception generates positive feelings about the surgery, and the patients are calm. The only fear shown on the subject relates to the fear of anaesthesia.

“I’m going calmly. I’m calm; it’s not an important operation” (P6, M, 65 yrs.).

##### The fear of not solving

Despite the expressed optimism about the type of surgery, patients show post-surgery fear.

The biggest fear is that of not resolving, not being able to return to everyday life and not improving the limitations from which they are suffering. This thought generates profound uncertainty. Most interviewees give significant importance to postoperative rehabilitation and plan how to deal with that period: wearing the brace for a month, taking a month of almost complete rest and paying close attention to movements.

“Rehabilitation is important because if I don’t do the rehabilitation well, the surgery will not be as good as it should be” (P1, F, 67 yrs.).

## Discussion

Our themes and sub-themes of the phenomenological analysis show that most patients undergoing to RCR described acute pain, severely disturbed sleep, limitation or inability to move, dependence on others for basic needs, and fear of not being solved. The negative impact of an RCT on the activities of daily life, work and adaptation strategies put in place to continue activities as normally as possible were analysed. Optimism and confidence for arthroscopic surgery were emphasised.

To our knowledge, the current study explores a topic that does not have extensive supporting literature. Minns Lowe CJ et al. described people’s experiences with symptomatic RCT, their symptoms, the impact on their daily life, and the coping strategies used by patients [14]. However, the study by Lowe and colleagues was limited to patients with severe medical conditions and in hospitals, and this data could alter the quality-of-life results. On the contrary, the present study involved a more heterogeneous sample size. Participants were RCR candidates but were enrolled regardless of injury severity. This allowed us to obtain more robust and even more generalisable results.

The present study’s first main and most relevant theme is “Pain changes lifestyle habits”. Pain is the most common and disabling symptom in all patients with symptomatic shoulder disease [3–5, 13]. The pain condition produces restriction or inability to move and impaired sleep quality in patients, generating a state of incompleteness, insufficiency, mortification, and suffering. The only desire is to regain all the former functionalities. The analysis of the experience of pain related to RCT in association with the study of Patient-Reported Outcome Measures can be used for evaluation as tools for assessing the quality of care and the state of health of patients and suggest important information to calibrate educational-therapeutic interventions.

The second theme is “Dependence on basic needs”. Care dependence can be related to suffering and humiliation [28]. The patients interviewed express intense suffering and humiliation depending on someone, even in their daily lives (e.g., washing, dressing, eating, etc.). Much concern derives from the postoperative thought that it could be even more disabling than the limitations that patients experience during data collection. It isn’t easy to find the availability of someone who can assist them. A thorough evaluation of patients’ experiences of addiction is essential to improving care and reducing patient suffering [9, 13, 14].

The third main theme is “Suffering turns time into waiting”. Not all patients have an acute and traumatic history of the disease. Sometimes the pain is described as a nuisance because it derives from chronic conditions. Therefore, with some effort, patients manage to carry out most of their daily activities, even work and underestimate the problem, believing that they can carry on with life, and they end up exhausted. Due to the pressing limitations they suffer daily, physically and mentally, they do not realise that surgery is the only solution. In this phase, patients experience a climate of anticipation and profound uncertainty.

The final central theme that emerged is “Optimism and confidence for arthroscopic surgery”. Evidence shows that arthroscopic RCRs improve patients’ quality of life and related disorders such as sleep [1, 29, 30]. The outcomes of rotator cuff surgery mainly depend on several factors, including tendon biology, surgical technique and comorbidities of the patient [31, 32]. Despite the decision to undergo surgery because they’re exhausted by the limitations related to painful symptoms, patients initially showed anxiety and fear of the surgery and anaesthesia. In reality, after having been to the pre-admission session with the orthopaedic surgeon, the prospect of arthroscopic surgery with all the related advantages seemed to generate positive feelings of optimism and confidence in the patient’s reduced hospitalisation and recovery times.

The limitations of this study could be due to the generalisation of the results. As the study was conducted in Italy with Italian participants, countries with other cultures and habits could present differences. Therefore, future research should explore whether and how patients’ experiences change after RCR, even retrospectively, to gain a broader view. This knowledge could support the development of specific educational and therapeutic strategies to improve care.

## Conclusions

The study showed, through the experiential analysis of the experiences of the patients interviewed, the negative impact of RCT on different aspects of daily life. The topics most discussed in the interviews are acute pain, severely disturbed sleep, limitation or inability to move, dependence on others for basic needs, and fear of not resolving the problem. Arthroscopic surgery appears to significantly impact the uncertainty experienced by patients waiting for surgery. Patients who become aware of this type of intervention tend to be calmer and more confident about a faster recovery.

The experiences described in the study could offer helpful insights for patient preoperative assessment and help healthcare professionals analyse and understand the power of the emotional, personal and social impacts of symptoms on RCT patients. This would ensure that patients needing help for emotional conditions such as fear, depression or extreme addiction are identified early and appropriate support, education and treatment are ensured.

## Data Availability

The datasets used and/or analysed during the current study are available from the corresponding author on reasonable request.

## Abbreviations

RCRSP: Rotator Cuff Related Shoulder Pain
RCT: Rotator Cuff Tear
VAS: Visual Analog Scale
RCR: Rotator Cuff Repair
